# Remote‐controlled genetics: A new frontier in precision therapy

**DOI:** 10.1002/ctm2.70553

**Published:** 2025-12-15

**Authors:** Zhihua Lin, Martin Fussenegger

**Affiliations:** ^1^ Department of Biosystems Science and Engineering ETH Zurich Basel Switzerland; ^2^ University of Basel Faculty of Life Science Basel Switzerland

## 1 CONVERGENCE OF SYNTHETIC BIOLOGY AND REMOTE‐CONTROL MODALITIES

**FIGURE 1:**
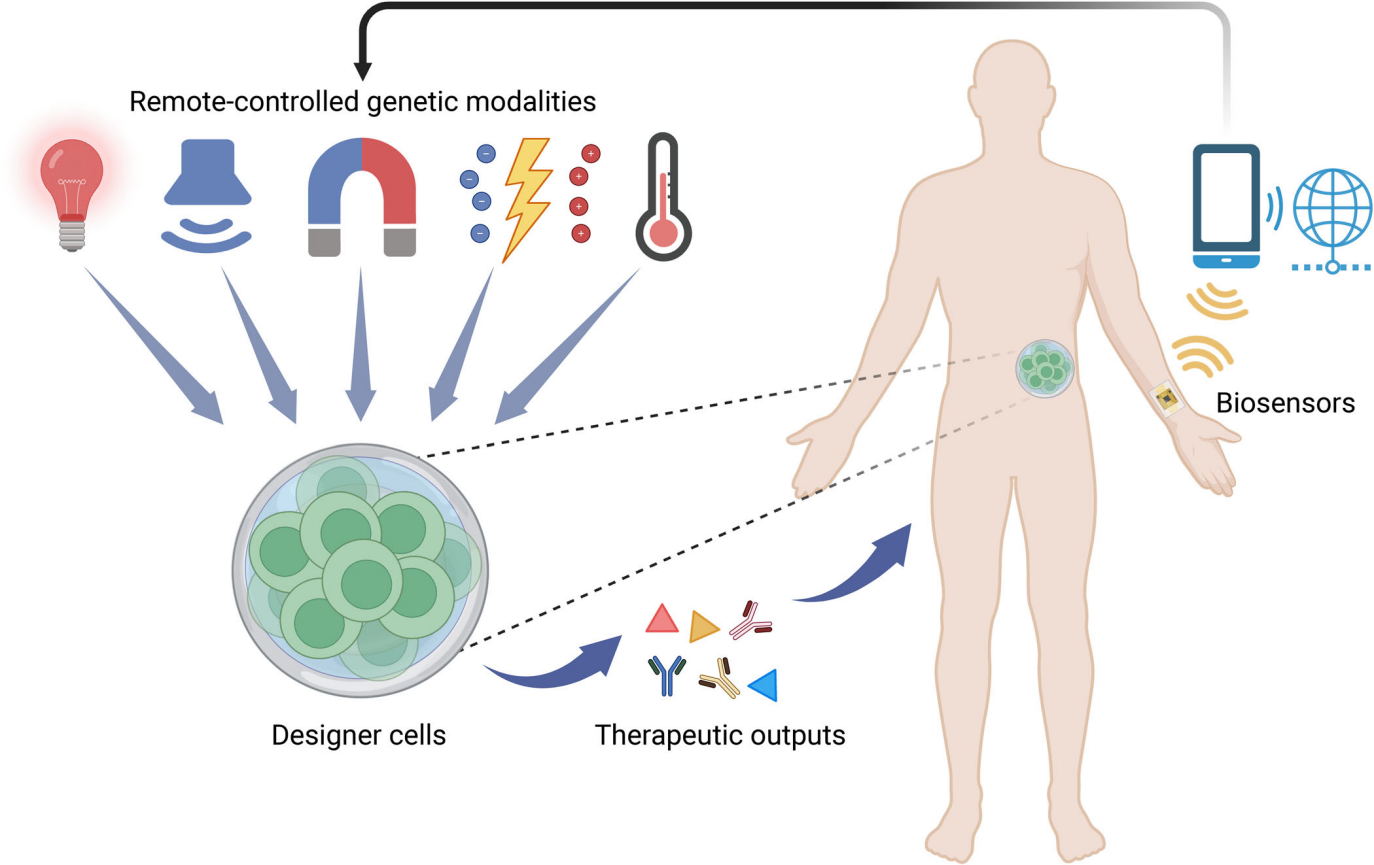
Illustration of an integrated remote‐controlled therapeutic system. Physical energy inputs − including optical, acoustic, magnetic, electrical, and thermal cues − activate engineered gene circuits within designer cells to produce programmable therapeutic outputs. Concurrently, wearable or implantable biosensors acquire real‐time physiological signals, forming a closed feedback loop that supports responsive patient‐specific control of gene‐based interventions. Created in BioRender. Lin, Z. (2005) http://BioRender.com/3vm7llr.

Next‐generation cell and gene therapies are expected to provide individualized treatment based on continuous monitoring of appropriate disease markers coupled with precise spatiotemporal regulation of therapeutic delivery. Compared to conventional therapies relying on chemical inducers, which afford limited ability to modulate dose and timing, remote‐controlled genetics is expected to be transformative, enabling real‐time, reversible, localized and patient‐specific control of genetic and cellular activity by coupling engineered biological systems with external physical triggers—light, ultrasound, magnetic fields, electrical currents or heat—to drive gene expression or protein secretion on demand (Figure 1). In these systems, physical signals serve as a wireless communicator, activating gene switches through transducers such as natural responsive proteins or protein‐protein interactions, responsive nanoparticles, polymeric scaffolds or hydrogels. Here, we briefly summarize available physical modalities, actual and potential applications, and prospects and challenges for the future.

## 2 PHYSICAL INPUT MODALITIES FOR REMOTE‐CONTROLLED GENETICS

The currently available technologies include optogenetics, sonogenetics, magnetogenetics, electrogenetics and thermogenetics. (i) Optogenetic platforms provide millisecond precision through light‐responsive transcription factors and ion channels or pumps,^1^ but offer limited tissue penetration. However, recent advances in far‐red and near‐infrared sensitive systems^2^ have expanded the range of available light‐sensitive proteins and also improved the tissue penetration depth. (ii) Sonogenetic systems typically use focused ultrasound to enable deep‐tissue stimulation of mechanosensitive or heat‐responsive circuits. Notably, however, music has recently been used for gene circuit activation via bacterial large conductance mechanosensitive channels introduced into mammalian cells.^3^ (iii) Magnetogenetic systems employ nanoparticles or native magnetic receivers (e.g., ferritin) that convert alternating magnetic fields into localized thermal, mechanical, or chemical signals in native biological environments.^4^ Recently, magnetoelectric nanoparticles have extended this concept by directly translating low‐frequency alternating magnetic fields into intracellular electrical stimuli, using local biosafe increases in reactive oxygen species to activate gene circuits.^5^ (iv) Electrogenetic systems bridge living tissues with implantable hardware by integrating cells with electrodes or conductive polymers, linking direct current (DC) electrical stimulation to redox signalling pathways,^6^ or using alternative current (AC) pulses to activate voltage‐responsive protein elements.^7^ (v) Thermogenetic systems combine miniatured extracorporeal and wireless electronic devices with a temperature‐sensitive promoter or protein thermometer to control gene expression via generation of mild hyperthermia.^8^

Collectively, these modalities establish a tunable “physical toolbox” for gene activation, offering clinicians the potential to provide reversible, on‐demand, multiple stimulations to meet diverse dosing needs while making it possible to balance depth, precision, and safety concerns according to therapeutic context. Their shared design logic is modular—physical input, biological interpretation, and independently optimized functional output—enabling flexible adaptation across diseases. Gene expression can be quantitatively regulated by adjusting two principal tunable parameters, intensity (or field amplitude) and frequency. Thus, these modalities provide tunable, wireless and reversible control over gene expression without chemical inducers, thereby minimizing pharmacokinetic variability and systemic toxicity.

## 3 THERAPEUTIC APPLICATIONS

Cells engineered with gene circuits responsive to remote triggers have been employed as living bio‐pumps to treat metabolic disorders such as diabetes, releasing therapeutic proteins such as insulin as required and eliminating the need for external pumps or continuous infusions.^1^, ^3^, ^5^, ^6^, ^7^

In oncology, responsive gene circuits can be engineered into immune cells or tumour‐targeting implants to produce antitumor cytokines, pro‐apoptotic factors, or checkpoint modulators when externally triggered, enabling programmable cytotoxic, immune‐activating, or pro‐apoptotic signals only when and where clinicians choose to trigger them, minimizing off‐target toxicity and enabling highly personalized intervention.^9^

Neurological disorders are also a compelling target. Ranging from light‐driven neural stimulation to magnetic‐electric stimulation of deep brain circuits,^10^ remote‐controlled stimulations provide minimally invasive alternatives to electrode implants for epilepsy, Parkinson's disease or depression. The integration of remote‐controlled gene circuits into neurons or transplanted therapeutic cells is expected to facilitate the regulation of neurotransmitter synthesis, neuromodulator release, or neuroprotective factor expression, transforming neural therapeutics from global, fixed‐dosed interventions into local, dynamic, or even programmable neuromodulation for targeted and personalised control over neural circuit activity and neuroregenerative processes.

Regenerative medicine increasingly relies on precise control of cellular behaviour to promote tissue repair, and remote‐controlled genetic systems offer powerful means to orchestrate these processes in two and three dimensions.^11^ Engineered stem cells, progenitors or supportive stromal cells can be equipped with remote‐controlled genetic systems to produce growth factors, cytokines, or extracellular matrix components. Such on‐demand systems have the potential to overcome limitations of traditional stem cell therapies, including poor engraftment, uncontrolled differentiation and transient paracrine signalling.

## 4 PERSPECTIVES

The advent of remote‐controlled genetics provides a basis for globally responsive therapeutic ecosystems, where engineered cells, bio‐chemical sensors and external devices form an integrated network that continuously interprets and responds to metabolic and physiological signals. We can envisage a future where wearable glucose monitors, lactate biosensors, transdermal ultrasound patches, magnetoelectric stimulators or soft electrophysiological skins stream continuous metabolic data to AI models,^12^ which then compute optimized stimulation patterns, such as light pulses, magnetic fields, ultrasound bursts or electrical commands, to activate therapeutic gene circuits pre‐positioned in specific tissues, facilitating precise interventions that match the complexity of whole‐body physiology. For example, such systems could stabilize impaired systemic homeostasis in various metabolic syndromes. Such a networked architecture could also interface with established cell therapy platforms, such as CAR‐T cells, CAR‐macrophages, engineered NK cells, and MSC‐based regenerative therapies, outfitted with remotely controlled gene switches to enable on‐demand or algorithmically triggered control over cell proliferation, cytokine release, killing activity, or persistence, reducing the risk of cytokine release syndrome and minimizing systemic toxicity.

Achieving this vision requires seamless integration between biological and electronic systems. For chronic interrogation and actuation of therapeutic cells, soft implantable optoelectronics, magnetoelectric stimulators, localized ultrasound arrays, and bioresorbable electrodes are needed. The utilization of AI‐assisted control algorithms to maintain physiological parameters within predefined limits necessitates the acquisition of patient‐specific data, encompassing fluctuations in metabolism, inflammation, tumour markers and neural activity. Such closed‐loop, bio‐digital feedback systems would represent a dramatic shift from the current model of episodic dosing to continuous, adaptive therapeutic governance, where living cells function as the primary executors of therapeutic tasks, while electronic components and artificial intelligence systems orchestrate the timing and address safety concerns.

However, such convergence faces major challenges before clinical translation. First, the electronic–biological interfaces are required to be biocompatible, stable, and power‐efficient over therapeutically relevant periods. All physical inputs (optical, magnetic, acoustic, thermal or electrical) must remain within safe exposure limits while reliably transducing signals into genetic responses. Further, as cell therapies become connected to networked external devices, issues of data privacy, cybersecurity, and potential bio‐digital interference arise. Wireless stimulators, wearable sensors, and cloud‐based AI pipelines are susceptible to the same vulnerabilities as medical IoT devices, and effective protection against unauthorised access, signal spoofing or “biohacking” is essential. Regulatory frameworks must evolve to evaluate the genetic constructs, digital infrastructure, encryption standards and cybersecurity practices associated with remote‐controlled therapies. Nevertheless, adaptive systems that learn from the patient's physiology, compute therapeutic responses and deliver molecular interventions autonomously offer the exciting prospect of precise, adjustable and personalized therapy. We believe the coming decade will likely redefine therapeutic practice—not through a single breakthrough, but through the integration of living cells, materials and machines into coherent, intelligent systems that can “think” and heal in real time.
